# Vascularization Strategies in Bone Tissue Engineering §

**DOI:** 10.3390/cells10071749

**Published:** 2021-07-11

**Authors:** Filip Simunovic, Günter Finkenzeller

**Affiliations:** Freiburg University Medical Center, Department of Plastic and Hand Surgery, Faculty of Medicine, University of Freiburg, 79106 Freiburg, Germany; guenter.finkenzeller@uniklinik-freiburg.de

**Keywords:** vascularization, tissue engineering, bioprinting, bone, mesenchymal stem cell, endothelial cell

## Abstract

Bone is a highly vascularized tissue, and its development, maturation, remodeling, and regeneration are dependent on a tight regulation of blood vessel supply. This condition also has to be taken into consideration in the context of the development of artificial tissue substitutes. In classic tissue engineering, bone-forming cells such as primary osteoblasts or mesenchymal stem cells are introduced into suitable scaffolds and implanted in order to treat critical-size bone defects. However, such tissue substitutes are initially avascular. Because of the occurrence of hypoxic conditions, especially in larger tissue substitutes, this leads to the death of the implanted cells. Therefore, it is necessary to devise vascularization strategies aiming at fast and efficient vascularization of implanted artificial tissues. In this review article, we present and discuss the current vascularization strategies in bone tissue engineering. These are based on the use of angiogenic growth factors, the co-implantation of blood vessel forming cells, the ex vivo microfabrication of blood vessels by means of bioprinting, and surgical methods for creating surgically transferable composite tissues.

## 1. Introduction

Bone can be divided into an external layer, named cortical bone, and an internal layer, referred to as cancellous bone. Cortical bone shows extreme mechanical stiffness with relatively low porosity [1,2]. In contrast, cancellous bone shows very high porosity and only about 10 percent of the mechanical stiffness of cortical bone. Within the cortical bone, osteons form functional units that have the so-called haversian canals in the center, which contain nerves and blood vessels [3,4]. These osteons with the associated haversian canals are not found in cancellous bone and are also not necessary for the blood vessel supply due to the high porosity of this bone compartment.

During embryonic development, bone can be formed in two different ways: enchondral ossification and intramembranous ossification [5]. Intramembranous ossification is typical for flat bones such as skull bone [6]. Here, osteoblastic precursor cells (mesenchymal stem cells) differentiate directly into osteoblasts, which then synthesize the corresponding bone matrix. The development of long bones, on the other hand, takes place via the process of enchondral ossification [7]. Here, mesenchymal stem cells initially differentiate into chondrocytes, which initially form a cartilaginous precursor of the bone. In the further course, the ingrowth of blood vessels into the cartilage causes its destruction. Invading mesenchymal stem cells then differentiate into osteoblasts and induce bone formation, as in intramembranous ossification. Vascularization is of crucial importance in both ossification processes as well as further bone formation and in bone remodeling processes. In this context, vascularization is not only necessary for the immigration of mesenchymal stem cells, but also, in particular, for the supply of the metabolically active bone tissue with oxygen and nutrients and removal of waste products.

The vascular endothelial growth factor (VEGF) plays a decisive role in the initiation of vascularization. It is formed by hypertrophic chondrocytes and mesenchymal stem cells and, as a potent angiogenic growth factor, induces the sprouting of preexisting surrounding blood vessels into the bone tissue [8]. It can therefore be assumed that VEGF is the decisive factor for the coupling of angiogenesis and osteogenesis. In mouse experiments, it could be shown that the inhibition of the VEGF function has a negative effect on bone angiogenesis and, thus, a negative effect on bone formation [9].

When a bone is fractured and blood vessels are damaged, hematoma forms and inflammatory cells migrate into the fracture site. The ingrowth of new blood vessels is essential for the formation of a soft callus consisting of fibroblasts and chondrocytes. The soft callus is, by way of enchondral ossification, converted into a rigid, calcified hard callus. Further mineralization and remodeling processes within the callus eventually lead to the repair of the damaged bone [10]. Here, too, it could be shown that VEGF plays an important role. For example, in a rabbit model, the application of recombinant VEGF to the fracture area led to an improvement in neovascularization and bone healing [11]. In contrast, the inhibition of VEGF in a mouse model resulted in decreased angiogenesis, decreased callus mineralization, and an inhibition of bone healing [12].

Due to the great importance of vascularization in both bone formation and in bone healing, it is clear that this aspect must also be taken into account in the tissue engineering of bone tissue (BTE). In the clinical context, bone defects can be treated with autologous or allogeneic bone grafts, with autologous bone replacement being the gold standard. Critical-size bone defects can be caused by trauma, tumor, osteomyelitis, avascular bone loss, and non-union fracture, and are thus very common. Bone transplantation is performed in an estimated 2.2 million procedures yearly worldwide [13]. Smaller defects are bridged by non-vascularized bone grafts (e.g., iliac crest), while larger defects require pedicled or free tissue transfer [14,15]. However, the use of autologous bones is restricted due to the limited availability and due to the fact that an additional bone defect is created. In the case of allogeneic bone transplantation, additional limitations include possible rejection by the host and the possibility of disease transmission [16,17].

This review aims to provide an overview of the current vascularization strategies in the field of BTE. The various strategies discussed in this review article are summarized in Table 1.

## 2. Induction of Vascularization by Angiogenic Growth Factors

Angiogenesis represents a mechanism by which new blood vessels are formed from a preexisting vascular network. In contrast, vasculogenesis means that new blood vessels are formed de novo by endothelial progenitor cells. During embryogenesis, these cells differentiate to mature endothelial cells and form a primitive vascular plexus that further matures into a hierarchical network with vessels of different calibers [18]. Under physiological as well as under pathophysiological conditions such as the growth of solid tumors, angiogenesis is initiated by the expression and secretion of pro-angiogenic diffusible soluble growth factors (GFs). Numerous pro-angiogenic growth factors have been identified in recent decades [19]. Prominent representatives are members of the vascular endothelial growth factor (VEGF) family [20], fibroblast growth factor (FGF) [21,22], angiopoietins (Ang) [23], transforming growth factor (TGF) [24,25], platelet-derived growth factor (PDGF) [26,27], and some interleukins [28,29]. Under normal conditions, the vasculature in the adult human body is relatively quiescent. However, during wound repair as well as in the context of solid tumor growth, angiogenesis in the respective tissues is dramatically stimulated. Angiogenic growth factors are expressed and secreted by a great variety of different cell types in response to different stimuli. Expression of VEGF, for example, can be induced by other GFs [30], activated oncogenes [31,32], and tissue hypoxia [33]. After secretion of the angiogenic GFs, they bind to their respective receptors on the cell surface of endothelial cells in neighboring blood vessels and induce several characteristic angiogenic cell responses. In order to trigger angiogenesis from neighboring vessels, the extracellular matrix and basement membrane of the blood vessel have to be degraded. This is achieved by inducing the expression of matrix metalloproteinases in endothelial cells. In addition, angiogenic GFs also induce the migration, invasion, and proliferation of endothelial cells, finally leading to the formation of a new lumen-containing blood vessel capillary, which matures by recruitment of vessel stabilizing mural cells such as pericytes or smooth muscle cells [34].

In order to induce in vivo neovascularization in tissue engineering applications, angiogenic GFs were mixed in hydrogels or coated on solid scaffolds that served as drug-release systems upon implantation. For example, Minardi and colleagues used multiscale microspheres, composed by a nanostructured silicon multistage vector (MSV) core and a poly(dl-lactide-co-glycolide) acid (PLGA) shell for controlled release of the angiogenic factor PDGF-BB [35]. The release kinetics of PDGF-BB was sustained over two weeks and a robust neovascularization was induced upon subcutaneous implantation in mice. Similarly, a VEGF release system was established by Chen and colleagues [36]. In this system, heparin cross-linked demineralized bone matrices were loaded with VEGF. This scaffold also showed sustained release of the angiogenic GF and induced angiogenesis in a subcutaneous implantation model. Angiogenic GFs were also used to induce vascularization in critical-sized bone defects. For example, Quinlan and colleagues incorporated VEGF in alginate microparticles [37]. These particles were then incorporated in collagen-hydroxyapatite scaffolds and implanted in a rat calvarial defect model. In these experiments, VEGF showed a sustained release rate and improved vascularization as well as bone formation in bone defects. The importance of VEGF in bone tissue engineering and bone regeneration and its central role in the coupling of angiogenesis and osteogenesis is highlighted by several recent review articles [38,39,40].

A great variety of natural [41,42,43], semi-synthetic [44,45], and synthetic [46,47,48,49] hydrogels have been used during the past decade for the release of a great variety of different GFs in order to improve neovascularization in BTE. From these studies, it became clear that an effective angiogenic therapy is strictly dependent not only on the right GF or the right combination of different GFs, but also on a temporal and dose controlled release of the angiogenic GFs. In the case of VEGF, for example, it was shown that a high initial burst of release, leading to an unphysiologically high concentration of VEGF in the implanted construct and the surrounding tissue, is associated with the appearance of functionally impaired blood vessels. In this case, the high concentration of VEGF leads to the formation of immature leaky blood vessels, whereas lower doses of VEGF induced the formation of mature functional blood vessels within the implanted construct [50].

As further development of the vascularization strategy on the basis of single recombinant angiogenic GFs, release systems were developed in which two or more GFs are released. For example, two angiogenic GFs, VEGF, and PDGF-BB were used with the aim to induce the formation of blood vessels with increased inherent stability and maturity [45,51]. Angiogenic GFs have also often been combined with osteogenic factors in order to enhance bone tissue formation in BTE applications. For example, Suárez-González and colleagues have loaded porous beta tricalcium phosphate (β-TCP) scaffolds with VEGF and a biologically active peptide derived from human bone morphogenetic protein (BMP)-2 [52]. They were able to show a controlled sequential delivery of both factors over a time course of 60 days. Moreover, by using a sheep intramuscular implantation model, in vivo biological activity of both factors was demonstrated. Similarly, Kuttappan and colleagues combined BMP-2 with VEGF or bFGF and were able to show increased vascularization and bone formation in a rat critical sized calvarial defect model [53].

Although many studies have shown significant benefits of angiogenic GF delivery, therapeutic effects are sometimes hampered by the short half-life of recombinant GFs and, as above-mentioned, by problems in achieving the correct spatial and temporal release kinetics. In summary, an effective and safe angiogenic therapy based on recombinant GFs is not only dependent on the right combination of GFs delivered, but also on a temporal and dose controlled release.

Therefore, alternative delivery strategies have been developed based on the gene modification of cells that are implanted into scaffolds for BTE. In many cases, osteogenic cells such as primary osteoblasts or mesenchymal stem cells (MSCs) were embedded in hydrogels and used for seeding solid scaffolds for implantation in bone defects. These cells can be genetically modified ex vivo with genes encoding angiogenic GFs before implantation. This results in transient or stable expression of the respective factors, depending on the method that is used to deliver the recombinant DNA into the cells. For example, Peng and colleagues genetically engineered muscle-derived stem cells to overexpress BMP-4 and VEGF [54]. By using a mouse calvarial bone defect model, they were able to show that BMP-4 and VEGF act synergistically to support bone healing. Similarly, Lee et al. transfected adipose tissue-derived stem cells (ASCs) with BMP-2 or VEGF encoding plasmids ex vivo [55]. By mixing the respective transfected ASCs in a ratio of 9:1 (BMP-2-transfected versus VEGF-transfected ASCs), they were able to show optimal effects on angiogenesis, osteogenesis, and bone healing after implantation of the cell mixture into a calvarial defect model. FGF was also used successfully in an ex vivo gene therapeutic approach to support bone healing [56]. In this case, FGF-2 was transfected into bone marrow-derived MSCs (BMSCs) before implantation in a rat calvarial defect. The authors were able to demonstrate increased vasculogenesis as well as improved bone healing in this defect model.

## 3. Cell-Based Strategies for Vascularization

Osteogenesis and vasculogenesis are key processes in the generation of bone tissue, and it is well established that they share common signaling pathways [57,58,59]. Therefore, co-culturing vascular and osteogenic lineage cells have become a cornerstone in BTE. Liu et al. were able to identify 259 publications on co-culture systems in tissue engineering, 85 of which focused on bone tissue [60]. This chapter focuses on cell types used for co-culture experiments, experimental models, and modes of communication between osteogenic and vasculogenic components.

### 3.1. Cell Types Used in Co-Culture Systems

Cells of various sources, differentiation stages, and from various species have been successfully used to support bone regeneration. Osteogenic cells can be categorized in mature primary osteoblast cultures, osteogenic precursors, cell lines, and bone marrow aspirates. Human osteoblast (hOB) primary cultures are widely employed and can be gained (e.g., from the femora of patients during hip arthroplasty). These cells are robust but they tend to proliferate slowly and cannot be expanded for more than several passages. The advantages are that they are a relevant clinical model, are able to produce mineralized bone matrix immediately upon implantation, and are able to induce angiogenesis. However, reproducing results with hOBs can prove challenging since patients are heterogeneous and, even in one patient, the osteoblasts are not a homogenous population. More commonly, immortalized bone cell lines are used because of ease of handling and better reproducibility of results [61]. However, these cell types have less clinical relevance and are not an adequate replacement for primary cultures, not least because immortalization changes the properties of the cells.

Bone marrow-derived osteoprogenitors were the first cell population to be used in BTE [62,63], but the quantity and quality of bone progenitors are highly variable. MSCs have multiple advantages and are under increased scrutiny for tissue engineering applications. MSCs are adult multipotent stem cells capable of differentiating in osteoblasts, chondrocytes, and adipocytes. MSCs have now been isolated from many sites throughout the body. They are primarily found in bone tissue, but since they are also present in adipose tissue, muscle, and the dental pulp, MSCs can be isolated with relative ease from the adult organism. The quality and the differentiation potential of MSCs vary and are subject to an increasing amount of research [64]. Based on the simplicity of isolation and their relatively high proliferation potential ex vivo, MSCs are very attractive for BTE application. However, upon implantation, it was recognized that these cells sometimes show limited survival and osteogenic potential, restricting their therapeutic value. In this context, it was shown that hypoxic preconditioning is beneficial for MSCs. Hypoxia induces Hif-1alpha-dependent expression of oxygen-sensitive genes such as VEGF and bFGF [65] and it can be assumed that this leads to improved angiogenesis upon implantation of preconditioned MSCs. Moreover, it was also shown that hypoxic preconditioning improves cell survival and osteogenic differentiation of MSCs, leading to improved bone healing in critical-sized bone defects upon implantation of preconditioned MSCs [66,67].

In BTE applications, the MSCs need to be directed toward osteogenic phenotype, most commonly through the addition of growth factors (see previous chapter) or through cultivation on bone-derived ECM, which is proven to have osteogenic (and vasculogenic) properties [68,69,70,71,72,73,74,75]. Alternative stem cell sources are embryonic stem cells and induced pluripotent stem cells [76].

Endothelial cells are a more heterogeneous family than osteoblasts. Endothelial cells (ECs) are different in arteries, veins, and capillaries, as are ECs from different organs, and ECs are found in micro- and macrocirculation. They differ with respect to morphology, density, permeability of the vessels, gene expression profile, hemostatic competence, and angiogenic potential [77]. The most commonly used ECs, due to ease of harvest and culturing, are human umbilical vein ECs (HUVECs). Since the vasculature required to perfuse the emerging bone construct is microvasculature, these are not the most physiological choice. However, harvesting microvascular ECs from bone or other organs is technically demanding, with the exception of dermal microvascular ECs. Endothelial progenitor cells (EPCs) are extracted from peripheral blood or umbilical cord blood. They have high proliferation potential and a higher capillary-forming potential compared to ECs [78]. Furthermore, they secrete osteogenic factors such as bone morphogenic protein family members, TGF-beta, and enhance osteogenesis [79].

### 3.2. Modes of Communication

Cells communicate through soluble molecules secreted in the extracellular space (paracrine signaling) by cell-to-cell contact (adherens junctions) or by direct cytoplasmic cell-to-cell transport of molecules through gap junctions. Signaling molecules can thus exert influence on both bone and vasculature forming cells if they are secreted by one of the partners.

Growth factors can be stored in the extracellular matrix (ECM) and, upon regulated proteolysis by matrix-metalloproteases, presented to cells [80,81,82]. This method of growth factor storage is so efficient that it has been possible to isolate growth factors from archeological findings dating from the Neolithic [83]. Additionally, components of the ECM can exert influence by direct mechanical action on the cellular membrane, which is transmitted inside the cell by the cytoskeleton. For example, integrins can trigger tyrosin-kinase receptors by direct contact [84]. The importance of ECM is becoming increasingly recognized. ECM of fibroblasts [85], chondrocytes, and endothelial cells [70,86] was shown to induce osteogenesis, and the ECM of osteoblasts induced the formation of the capillaries [87]. These observations are highly relevant for tissue engineering applications, as it is possible to seed ECM on a scaffold and thus eliminate the need for one co-culture partner.

Another possible mode of cellular communication is the exchange of microRNA molecules. During the past decades, a number of miRNAs have been identified that regulate osteogenesis and vaculogenesis, and recently, there has been interest by our group and others in identifying key miRNAs that influence both processes. Based on experimental data and proposed targets, the following miRNAs have been presumed to play a role in osteogenic–angiogenic coupling: miR-9, miR-10a, miR-20a, miR-26a, miR-29b, miR-31, miR-34a, miR-92a, miR-125b, miR-126 [88,89,90], miR-135b, miR-181a, miR-195, miR-200b, miR-210, miR-222, and miR-424 (see Frohlich et al. for an exhaustive review [91]). Identifying key miRNA players would be favorable as these can be effectively augmented or inhibited in in vitro and in vivo conditions.

### 3.3. Co-Culture Models

Under experimental conditions, cells can be co-cultivated with or without direct contact to each other. Furthermore, cell cultures can be 2- or 3-dimensional. A culture prohibiting cellular contact will investigate paracrine factors, and such experiments can be performed while separating cells by a membrane, or using conditioned medium or conditioned ECM. In performing co-culture experiments, the ratio of co-cultivation partners is important, as is the choice of medium, which needs to ensure viability and induce the desired functional development of both cell types. Cultivating cells separately will discard the need for separating cells and will provide a purer population for investigation. However, we and others have repeatedly observed that the strongest osteogenic and angiogenic effects are observed in direct contact [57,88,92,93].

A direct co-culture allows multiple levels of interaction between cells and is a more physiologic model. However, it requires that the cells are separated before performing experiments, and this implies a possibility of the contamination of results by the co-cultivation partner. Thus, the efficacy of the separation method has to be reliably and repeatedly tested. Most studies have been performed in 2D cultures, but cells can also be co-cultured as spheroids or within a scaffold as 3D cultures. 3D cultures are closer to physiological conditions and they allow for the investigation of more complex cellular behavior (e.g., tube formation by ECs) [57,59,94,95,96,97]. 3D cultures on scaffolds are closer to the construct, which is to be implanted in the in vivo part of a study than a 2D culture [98,99,100].

As discussed in other parts of this review, bone tissue engineering is making remarkable strides and we are now able to produce constructs which, upon implantation, form perfused blood vessels and produce mineralized matrix [101]. However, knowledge of common osteogenic and angiogenic processes, types of partners and conditions of co-cultivation as well as modes of interaction between them, are prerequisites for such developments, and these can be gained only by systematic and standardized research under defined conditions.

## 4. Induction of Vascularization by Biofabrication of Vessel Networks

The classical BTE concept is based on scaffolds that are randomly seeded with bone-forming cells and cells that provide the vasculature upon implantation, as already discussed in the previous sections. However, the prevascularization strategy that is based on the targeted fabrication of blood vessels employs the technique of 3D-bioprinting.

3D-bioprinting represents an additive manufacturing process for the production of artificial tissues that combines biomaterials, cells, and growth factors that are deposited in a layer-by-layer manner [102,103]. This technique allows precise control of the spatial distribution of cells within the bioprinted construct and opens the way to printing multiple cell types in a biomimetic manner to produce an artificial tissue that corresponds more closely to the native target tissue than would have been possible with classical tissue engineering.

### 4.1. Bioprinting Methods

Different printing methods have been adapted for the bioprinting of mammalian cells. These methods include inkjet printing (e.g., drop-on-demand, DoD), extrusion-based printing, and laser-assisted printing [104]. For bioprinting, cells are dispersed into hydrogels to form a so called bioink. The hydrogels need to exhibit high cytocompatibility, must be printable, and must provide a sufficient mechanical stability to the printed construct [105], where the integration of all these conditions is a major challenge in bioprinting.

Inkjet printing is a non-contact printing technique that could precisely deposit droplets of cell-laden bioinks. The diameter of the bioprinted droplets using cell-laden bioinks typically varies between 110 and 150 µm, depending on the cell concentration [106]. This technique has the advantage of high printing speed and high resolution, but is limited by the fact that only low-viscosity bioinks can be used for bioprinting. Extrusion-based bioprinting in contrast can use bioinks with a wide range of viscosities and represents a contact printing technique by which bioinks are extruded through a microscale nozzle. Due to its ease of control and cost-effectiveness, extrusion-based bioprinting is widely used, but is limited by a relatively low printing speed [107,108]. In contrast to extrusion-based bioprinting, laser-assisted bioprinting is relatively expensive, but is associated with a high printing resolution and a high post-printing cell survival [109].

The technology of bioprinting has already been used to print of blood vessels in 3D-tissue constructs and can be considered to represent a new kind of prevascularization strategy. There are two major routes of vascularization reported in the literature: the bioprinting of vessel-forming cells (endothelial cells) and the printing of sacrificial structures that can be removed, leaving behind a microchannel that can be populated by endothelial cells. Table 2 gives an overview of the vascularization strategies that are followed in bioprinting.

### 4.2. Bioprinting of Endothelial Cells

Endothelial cells are bioprinted in high density in suitable bioinks such as fibrin or collagen in the form of lines or branched structures in the form of native blood vessels. Upon in vitro culture of these bioprinted constructs, self-organization of endothelial cells and lumen formation can be detected. These endothelial structures resemble early blood vessel capillaries in such a way that lumina are confined by a single layer of endothelial cells.

Our group has bioprinted HUVECs in the form of high density suspension, or in the form of endothelial spheroids by drop-on-demand in fibrin hydrogels as bioink [100]. After three days of in vitro incubation, HUVECs formed a lumen-containing blood vessel with a single layer of CD31-positive endothelial cells. From this main vessel, smaller, undirected side branches were formed by sprouting cells, inducing a first step toward a simplistic hierarchically organized network. Exactly the same approach was used to print HUVECs by DoD inside a cuboid structure consisting of adipose tissue-derived MSCs that were printed by extrusion-based bioprinting. These constructs were implanted subcutaneously into SCID mice. Bioprinted HUVECs were able to form functional perfused blood vessels of different calibers in vivo. These human blood vessels were stabilized by mouse mural cells invading the artificial tissue. Moreover, in these experiments, bioprinted MSCs were able to synthesize a calcified bone matrix as an indicator of ectopic bone formation [101].

Similarly, Leucht and colleagues printed human dermal microvascular endothelial cells (HDMECs) and adipose tissue-derived MSCs in gelatin-based hydrogels by extrusion printing [111]. In this case, HDMECs were able to form vascular-like structures within 14 days of in vitro culture. Byambaa and colleagues also used extrusion–based bioprinting for the generation of vascularized structures [112]. In this case, HUVECs were printed in a gelatin methacryloyl (GelMA) hydrogel and were able to form capillary-like networks in vitro. Laser-assisted bioprinting was also used for printing ECs. Kérourédan et al. printed human endothelial progenitor cells (EPCs) along with MSCs between two collagen layers and observed the formation of a capillary-like vascular network, emerging from bioprinted EPCs [124]. The same group also used laser-assisted bioprinting for in situ printing of HUVECs. In this case, HUVECs were directly printed into critical-sized calvaria bone defects of mice, filled with MSC- and VEGF-loaded collagen. Bioprinted HUVECs were able to form organized microvascular networks on the collagen surface inside the bone defect, resulting in increased vascularization and bone regeneration rates after two months [125].

Extrusion-based bioprinting of HUVECs, along with MSCs in fibrin-hydrogels, was also used to print osteon-like patterns in vitro. In this case, the mechanical strength of the constructs was improved by additional printing of polycaprolacton (PCL), resulting in mechanical properties similar to native cortical bone. Upon subcutaneous implantation of the 3D-printed constructs, a significant increase in the number of blood vessels was detected in the 3D printed osteon-like scaffold [116].

HUVECs were also combined with other vascular cells such as smooth muscle cells (SMCs). For example, Xu and colleagues used extrusion-based bioprinting of HUVECs and SMCs in gelatin methacyloyl-based hydrogels (GelMA) with different concentrations of GelMA [117]. They produced a heterogeneous bilayer, composed of HUVECs-embedded in GelMA as an inner layer and SMC-loaded GelMA as an outer layer. The resulting heterogeneous bilayer construct closely resembled a native blood vessel.

### 4.3. Bioprinting of Microchannels

For the bioprinting of microchannels, sacrificial fugitive bioinks are used. These are printed in an environment of stable hydrogels containing the tissue forming cells. The sacrificial bioink is then removed by enzymatic, thermal, or mechanical methods or by incubation in cell culture medium, leaving behind hollow channels that can be populated by endothelial cells. The endothelial cells attach to the inner surface of the microchannels, thereby forming a uniform layer. In most cases, HUVECs are used as a source of endothelial cells. A classical sacrificial bioink that is often used is gelatin that can be printed by extrusion-based bioprinting at room temperature and can be liquefied by the incubation of the printed construct at 37 °C [127,129,131,142]. Examples of sacrificial bioinks that can be removed by enzymatic digestion are alginate and hyaluronic acid, which can be digested after printing by the incubation of the constructs in cell culture medium containing alginate lyase or hyaluronidase, respectively [128,141].

Another popular sacrificial bioink is Pluronic F127, a triblock copolymer that can be easily printed and removed under mild conditions [132,133]. For example, Kolesky and colleagues printed Pluronic F127 at room temperature in a surrounding of human dermal fibroblasts suspended in gelatin methacrylate(GelMA) that was photopolymerized by UV-light after printing [133]. After bioprinting, the constructs were cooled down to 4 °C to liquefy the printed Pluronic strands inside the construct and the Pluronic strands removed by the application of a vacuum, leaving behind open microchannels. Thereafter, HUVECs were injected into the microchannels where they formed a confluent monolayer within 48 h of incubation.

Although the goal of creating hollow microchannels is quite easy to achieve with the bioinks described above, numerous other bioinks have been described in this context. Other sacrificial bioinks that were used in the literature for the fabrication of hollow microchannels comprise polyvinylalcohol [135], gelatin-sucrose [140], carbohydrate glass [139], and agarose [137,138].

## 5. Surgical Strategies for Vascularization

A major issue with all the above strategies is the time needed until the replaced tissue is vascularized by the host. Tissues are oxygenized by diffusion over a distance of 200 µm, meaning that cells in the middle of the construct will invariably undergo hypoxic cell death [143,144,145]. In order to create stable constructs that reliably integrate in the host tissue, it is paramount to deliver nutrients to these cells by neovascularization. Neovasculariation can take the form of angiogenesis, which describes a process in which host vessels proliferate and penetrate the construct, or vasculogenesis, which implies that vessels are developed in the implant. In order to avert hypoxic cell death, which occurs during the first days after implantation [146], surgical strategies have been devised in order to vascularize the implant immediately after implantation.

The “in vivo bioreactor” principle unites traditional reconstructive surgery techniques and tissue engineering by relying on the host body as a bioreactor [147]. Imaging data are used to calculate the form and size of the defect. Chambers are then created, which correspond to the defect, filled with the preferred construct (cells, scaffold, growth factors), and implanted at a defect-remote site in the body. After a period of prelamination, the flap containing the construct is transferred into the defect and the vascular pedicle of the flap containing the construct is microsurgically anastomosed to recipient vessels. In this way, immediate and complete perfusion of the construct is achieved [143,148]. This technique is well-established experimentally in large animals (pig and sheep) and has been successfully performed in humans. In 2004, a subtotal replacement mandible consisting of a titanium mesh cage filled with bone mineral blocks coated in bone morphogenic protein 7 and bone marrow aspirate containing undifferentiated precursors was grown inside the latissimus dorsi muscle of a patient with previous subtotal mandibulectomy due to neoplasm. The muscle-construct unit was microsurgically transferred to the recipient site after seven weeks, and it showed good integration and a favorable aesthetic and functional outcome [149]. This approach avoids raising bone donor tissue, but it necessitates raising a muscle flap, which also represents considerable donor site morbidity. Another drawback is the technical complexity, need for two major surgeries, and a treatment period of several months.

Alternatively, and in order to avoid the harvest of a flap, an arteriovenous (AV) loop can be created. This approach involves anastomosing an artery with a vein, and transferring this “neopedicle” to the construct chamber, where it has to undergo microvascular sprouting in order to infiltrate the construct [150]. This approach was extensively verified experimentally [151,152] in large animal models [153] and clinically [154]. This technique also requires considerable time for the engineered tissue to vascularize by sprouting, necessitating hospitalization of several months [155], and thus limiting its clinical use.

To accelerate vascularization in the AV-loop chamber, growth factors [156] and stem cells [157] have been incorporated in the construct with promising results. These data suggest that combining other vascularization strategies in an AV-loop concept is a promising avenue of further research.

## 6. Conclusions and Future Directions

Strategies to support vascularization play an important role in the tissue engineering of artificial tissues, especially bone tissue. In this review article, we focused on four different vascularization strategies. These are based on the use of angiogenic growth factors, the implantation of endothelial cells, microfabrication of blood vessels by means of bioprinting, and on microsurgical principles. Each of these strategies has its specific advantages and disadvantages. However, based on our literature review, it has become clear that (co)implantation of bone- and blood vessel-forming cells is currently the predominant strategy in in vivo bone healing models.

It is feasible that tissue engineering applications will increasingly enter clinical settings and commercial applications. A good example of a standardized protocol with large-scale potential is the production of scaffolds seeded by mesenchymal stem cells extracted from the host (www.epibone.com, accessed on 7 July 2021). The scaffolds are perfused in bioreactors that are tailored to the defect, based on computer tomography images [158].

Future developments will rely on improvements in one single strategy. Progress is to be sought in the combination of strategies. A thorough understanding of all the presented methods as well as the experimental and clinical aspects of the task at hand, is required to compensate for individual disadvantages of each strategy. Furthermore, access to novel 3D bioprinting technologies allows for the convergence of bioengineering, materials science, and cell biology at an unprecedented level. Specifically, it is now possible to create 3-dimensional constructs containing functioning vessels, multiple cell types, a rigid external scaffold, and an internal hydrogel containing recombinant growth factors.

## Figures and Tables

**Table 1:** Advantages and disadvantages of the various vascularization strategies.

Vascularization Strategy	Advantages	Disadvantages
Angiogenic growth factors	technically simple	optimization of drug-release system is necessary
	numerous angiogenic growth factors are commercially available	time delay between implantation and achieving sufficient blood supply of implant
	combination of angiogenic and osteogenic growth factors is possible	short half-life of some angiogenic growth factors
Cell-based strategies	prevascularization of vessel networks in the implant is possible by seeding endothelial cells	limited survival of implanted cells
	combination of endothelial cells and bone-forming cells is feasible	in a prospective clinical setting, autologous cells have to be used
Biofabrication of vessel networks	precise control of the spatial distribution of cells	limited survival of implanted cells
	combined printing of cells and drug release hydrogels is feasible	
	combined printing of endothelial cells and bone-forming cells is feasible	in a prospective clinical setting, autologous cells have to be used
	acceleration of vessel formation by spatial alignment of endothelial cells	
Surgical strategies	immediate blood supply can be achieved	technically challenging
	combination of immediate vascularization and naturally occurring vascular sprouting in the AV-loop model	two stage approach is necessary to create a vascularized implant
	integration of growth factors and cells is feasible in the AV-loop model	

**Table 2:** Strategies for the bioprinting of blood vessels: bioprinting of cells and bioprinting of channels.

Printing Method	Bioink/Sacrificial Ink	Cell Type in Bioink/Cell Type Used for Endothelialization of Microchannels	References
**Bioprinting of cells**			
inkjet	fibrin	HUVECs	[100,101]
inkjet	fibrin	HUVEC spheroids	[99]
inkjet	agarose/collagen	HUVECs, human dermal fibroblasts	[110]
extrusion	gelatin-based	human dermal microvascular endothelial cells	[111]
extrusion	gelatin methacryloyl (GelMA)	HUVECs	[112,113]
extrusion	gelatin methacryloyl (GelMA)/fibrin	HUVECs	[114]
extrusion	gelatin methacryloyl (GelMA)	human aortic endothelial cells (HAECs), human aortic smooth muscle cells	[115]
extrusion	fibrin/polycaprolacton (PCL)	HUVECs	[116]
extrusion	gelatin methacryloyl (GelMA)/hyaluronic acid (HA)/glycerol/gelatin	HUVECs, smooth muscle cells (SMCs)	[117]
extrusion	laponite/alginate/methylcellulose	HUVECs	[118]
extrusion	methacrylated hyaluronic acid/methacrylated gelatin/hyaluronic acid	stromal vascular fraction (SVF)	[119]
extrusion	collagen	dermal fibroblasts (FBs)/human endothelial colony-forming cells (HECFCs)/placental pericytes (PCs)	[120]
extrusion	alginate/PEG-fibrinogen	HUVECs	[121]
extrusion	catechol-functionalized gelatin methacrylate (GelMA/C)	HUVECs, human coronary artery smooth muscle cells (HCASMCs)	[122]
extrusion	hyaluronic acid/glycerol/gelatin/fibrin	HUVECs	[123]
laser-assisted printing	collagen	endothelial progenitor cells (EPCs)	[124]
laser-assisted printing	collagen	HUVECs	[125,126]
**Bioprinting of microchannels**			
inkjet	gelatin	HUVECs	[127]
inkjet	alginate	without cells	[128]
extrusion	gelatin	HUVECs	[129]
extrusion	gelatin	without cells	[130]
extrusion	gelatin	smooth muscle cells (SMC)	[131]
extrusion	Pluronic F127	HUVECs, smooth muscle cells (SMC), human dermal neonatal fibroblasts (HDF)	[132]
extrusion	Pluronic F127	HUVECs	[133]
extrusion	Pluronic F127	without cells	[134]
extrusion	polyvinylalcohol	without cells	[135]
extrusion (coaxial)	alginat	without cells	[136]
extrusion	agarose	HUVECs	[137,138]
extrusion	carbohydrate glass	HUVECs	[139]
extrusion	gelatin-sucrose	HUVECs	[140]
stereolithography	hyaluronic acid	HUVECs	[141]

## Data Availability

There is no supporting data pertinent to this article.
